# Supplementary material for: Modeling glioblastoma heterogeneity as a dynamic network of cell states
Source: Mol Syst Biol. 2021 Sep 16;17(9):e10105. doi: 10.15252/msb.202010105 (PMC8444284; doi:10.15252/msb.202010105)
Supplement: Supplementary file 6 — Source Data for Figure 5 [file MSB-17-e10105-s004.zip › Figure5A_sourcedata/GSEA_3071/nosigdb_stateVI.GseaPreranked.1622795169056/gsea_report_for_na_neg_1622795169056.html]

Report for na\_neg 1622795169056 [GSEA]

| GS  follow link to MSigDB | GS DETAILS | SIZE | ES | NES | NOM p-val | FDR q-val | FWER p-val | RANK AT MAX | LEADING EDGE || 1 | IVY\_CT | Details ... | 106 | -0.69 | -3.49 | 0.000 | 0.000 | 0.000 | 521 | tags=61%, list=14%, signal=69% |
| 2 | RICHARDS\_ZHONG\_NATURE\_2018\_OPC\_UPREG | Details ... | 50 | -0.72 | -3.11 | 0.000 | 0.000 | 0.000 | 514 | tags=66%, list=14%, signal=76% |
| 3 | RICHARDS\_NOWAKOWSKI\_SCIENCE\_2017\_OPC\_UPREG | Details ... | 185 | -0.56 | -3.00 | 0.000 | 0.000 | 0.000 | 578 | tags=45%, list=16%, signal=51% |
| 4 | SINGH\_SOX2\_UPREGULATED\_TRANSCRIPTS\_PMID\_28122245 | Details ... | 193 | -0.55 | -2.96 | 0.000 | 0.000 | 0.000 | 734 | tags=49%, list=20%, signal=58% |
| 5 | RICHARDS\_NOWAKOWSKI\_SCIENCE\_2017\_ASTROCYTE\_UPREG | Details ... | 275 | -0.53 | -2.94 | 0.000 | 0.000 | 0.000 | 584 | tags=42%, list=16%, signal=46% |
| 6 | RICHARDS\_NOWAKOWSKI\_SCIENCE\_2017\_MGE\_IPC2\_UPREG | Details ... | 284 | -0.51 | -2.86 | 0.000 | 0.000 | 0.000 | 1231 | tags=69%, list=34%, signal=97% |
| 7 | RICHARDS\_VERHAAK\_CANCERCELL\_2010\_PRONEURAL | Details ... | 65 | -0.62 | -2.83 | 0.000 | 0.000 | 0.000 | 994 | tags=75%, list=27%, signal=102% |
| 8 | LARSSON\_STATE\_6 | Details ... | 55 | -0.64 | -2.82 | 0.000 | 0.000 | 0.000 | 834 | tags=69%, list=23%, signal=88% |
| 9 | RICHARDS\_NOWAKOWSKI\_SCIENCE\_2017\_NIN1\_UPREG | Details ... | 82 | -0.59 | -2.82 | 0.000 | 0.000 | 0.000 | 754 | tags=60%, list=21%, signal=74% |
| 10 | RICHARDS\_NEFTEL\_CELL\_2019\_NPC1 | Details ... | 27 | -0.74 | -2.80 | 0.000 | 0.000 | 0.000 | 596 | tags=74%, list=16%, signal=88% |
| 11 | RICHARDS\_NEFTEL\_CELL\_2019\_AC | Details ... | 21 | -0.78 | -2.79 | 0.000 | 0.000 | 0.000 | 314 | tags=67%, list=9%, signal=73% |
| 12 | SUVA\_NPC1\_PMID\_31327527 | Details ... | 27 | -0.74 | -2.76 | 0.000 | 0.000 | 0.000 | 596 | tags=74%, list=16%, signal=88% |
| 13 | SUVA\_AC\_PMID\_31327527 | Details ... | 21 | -0.78 | -2.76 | 0.000 | 0.000 | 0.000 | 314 | tags=67%, list=9%, signal=73% |
| 14 | RICHARDS\_NOWAKOWSKI\_SCIENCE\_2017\_MGE\_IPC3\_UPREG | Details ... | 76 | -0.58 | -2.76 | 0.000 | 0.000 | 0.000 | 798 | tags=62%, list=22%, signal=78% |
| 15 | LARSSON\_STATE\_2 | Details ... | 54 | -0.63 | -2.73 | 0.000 | 0.000 | 0.000 | 899 | tags=67%, list=25%, signal=87% |
| 16 | LARSSON\_STATE\_4 | Details ... | 66 | -0.58 | -2.72 | 0.000 | 0.000 | 0.000 | 1006 | tags=73%, list=28%, signal=99% |
| 17 | GAROFANO\_IAVARONE\_LIST\_OF\_NEURAL\_PROGENITOR\_MARKER\_GENES | Details ... | 59 | -0.60 | -2.71 | 0.000 | 0.000 | 0.000 | 680 | tags=54%, list=19%, signal=66% |
| 18 | RICHARDS\_NOWAKOWSKI\_SCIENCE\_2017\_IN\_CTX\_CGE2\_UPREG | Details ... | 105 | -0.54 | -2.68 | 0.000 | 0.000 | 0.000 | 606 | tags=47%, list=17%, signal=54% |
| 19 | RICHARDS\_ZHONG\_NATURE\_2018\_ASTROCYTES\_UPREG | Details ... | 145 | -0.51 | -2.66 | 0.000 | 0.000 | 0.000 | 507 | tags=39%, list=14%, signal=43% |
| 20 | DOROSZKO\_U3054MG\_CLUSTER\_0 | Details ... | 46 | -0.62 | -2.64 | 0.000 | 0.000 | 0.000 | 629 | tags=59%, list=17%, signal=70% |
| 21 | TISIG\_PMID\_32649888 |  | 28 | -0.70 | -2.61 | 0.000 | 0.000 | 0.000 | 360 | tags=54%, list=10%, signal=59% |
| 22 | DOROSZKO\_PERIVASCULAR\_INVASION |  | 23 | -0.70 | -2.59 | 0.000 | 0.000 | 0.000 | 330 | tags=57%, list=9%, signal=62% |
| 23 | OUTERRADIALGLIA |  | 38 | -0.63 | -2.59 | 0.000 | 0.000 | 0.000 | 453 | tags=47%, list=12%, signal=54% |
| 24 | POLLEN\_OUTERRADIALGLIA\_PMID\_26406371 |  | 38 | -0.63 | -2.57 | 0.000 | 0.000 | 0.000 | 453 | tags=47%, list=12%, signal=54% |
| 25 | ALLEN\_112\_ASTROL1FGFR3MT1G |  | 15 | -0.79 | -2.56 | 0.000 | 0.000 | 0.000 | 311 | tags=67%, list=9%, signal=73% |
| 26 | RICHARDS\_NOWAKOWSKI\_SCIENCE\_2017\_VRG\_UPREG |  | 225 | -0.47 | -2.56 | 0.000 | 0.000 | 0.000 | 561 | tags=37%, list=15%, signal=41% |
| 27 | LARSSON\_STATE\_3 |  | 50 | -0.58 | -2.54 | 0.000 | 0.000 | 0.000 | 308 | tags=36%, list=8%, signal=39% |
| 28 | VANGRONINGEN\_ADRN |  | 147 | -0.48 | -2.52 | 0.000 | 0.000 | 0.000 | 953 | tags=57%, list=26%, signal=74% |
| 29 | FELDMAN\_2020\_M-EARLYG1 |  | 67 | -0.54 | -2.52 | 0.000 | 0.000 | 0.000 | 306 | tags=36%, list=8%, signal=38% |
| 30 | RICHARDS\_NEFTEL\_CELL\_2019\_OPC |  | 21 | -0.70 | -2.49 | 0.000 | 0.000 | 0.000 | 561 | tags=62%, list=15%, signal=73% |
| 31 | FELDMAN\_2020\_S |  | 91 | -0.50 | -2.46 | 0.000 | 0.000 | 0.000 | 890 | tags=59%, list=24%, signal=77% |
| 32 | GAROFANO\_IAVARONE\_PPR\_SUBTYPE |  | 194 | -0.45 | -2.46 | 0.000 | 0.000 | 0.000 | 1697 | tags=84%, list=47%, signal=148% |
| 33 | ALLEN\_113\_ASTROL1FGFR3FOS |  | 15 | -0.77 | -2.45 | 0.000 | 0.000 | 0.000 | 291 | tags=60%, list=8%, signal=65% |
| 34 | RICHARDS\_NOWAKOWSKI\_SCIENCE\_2017\_NIN5\_UPREG |  | 129 | -0.48 | -2.45 | 0.000 | 0.000 | 0.000 | 994 | tags=57%, list=27%, signal=75% |
| 35 | RICHARDS\_NOWAKOWSKI\_SCIENCE\_2017\_IPC\_NEN1\_UPREG |  | 130 | -0.49 | -2.44 | 0.000 | 0.000 | 0.000 | 608 | tags=41%, list=17%, signal=47% |
| 36 | RICHARDS\_NOWAKOWSKI\_SCIENCE\_2017\_NIN3\_UPREG |  | 86 | -0.51 | -2.43 | 0.000 | 0.000 | 0.000 | 585 | tags=42%, list=16%, signal=49% |
| 37 | OLSEN\_SCPLIKE |  | 48 | -0.57 | -2.43 | 0.000 | 0.000 | 0.000 | 599 | tags=46%, list=16%, signal=54% |
| 38 | NB\_KI\_CLUSTER5 |  | 52 | -0.55 | -2.42 | 0.000 | 0.000 | 0.000 | 1092 | tags=73%, list=30%, signal=103% |
| 39 | GAROFANO\_IAVARONE\_NEU\_SUBTYPE |  | 94 | -0.49 | -2.41 | 0.000 | 0.000 | 0.000 | 1128 | tags=65%, list=31%, signal=92% |
| 40 | SUVA\_OPC\_PMID\_31327527 |  | 21 | -0.70 | -2.41 | 0.000 | 0.000 | 0.000 | 561 | tags=62%, list=15%, signal=73% |
| 41 | RICHARDS\_NEFTEL\_CELL\_2019\_NPC2 |  | 25 | -0.65 | -2.40 | 0.000 | 0.000 | 0.000 | 596 | tags=64%, list=16%, signal=76% |
| 42 | DOROSZKO\_U3013MG\_CLUSTER\_0 |  | 20 | -0.69 | -2.40 | 0.000 | 0.000 | 0.000 | 235 | tags=55%, list=6%, signal=58% |
| 43 | NB\_KI\_CLUSTER4 |  | 55 | -0.53 | -2.38 | 0.000 | 0.000 | 0.000 | 1177 | tags=75%, list=32%, signal=109% |
| 44 | SUVA\_NPC2\_PMID\_31327527 |  | 25 | -0.65 | -2.36 | 0.000 | 0.000 | 0.001 | 596 | tags=64%, list=16%, signal=76% |
| 45 | DOROSZKO\_U3180MG\_CLUSTER\_2 |  | 215 | -0.43 | -2.35 | 0.000 | 0.000 | 0.002 | 799 | tags=43%, list=22%, signal=52% |
| 46 | TIISIG\_PMID\_32649888 |  | 17 | -0.71 | -2.35 | 0.000 | 0.000 | 0.002 | 524 | tags=59%, list=14%, signal=68% |
| 47 | OLSEN\_ADRENERGIC |  | 42 | -0.57 | -2.35 | 0.000 | 0.000 | 0.002 | 601 | tags=52%, list=17%, signal=62% |
| 48 | RICHARDS\_NOWAKOWSKI\_SCIENCE\_2017\_IN\_CTX\_MGE1\_UPREG |  | 143 | -0.46 | -2.34 | 0.000 | 0.000 | 0.002 | 955 | tags=51%, list=26%, signal=66% |
| 49 | RICHARDS\_NOWAKOWSKI\_SCIENCE\_2017\_MGE\_DIV\_UPREG |  | 312 | -0.42 | -2.34 | 0.000 | 0.000 | 0.002 | 1310 | tags=63%, list=36%, signal=89% |
| 50 | RICHARDS\_NEFTEL\_CELL\_2019\_G1\_S |  | 20 | -0.65 | -2.28 | 0.000 | 0.000 | 0.004 | 1086 | tags=90%, list=30%, signal=128% |
| 51 | FELDMAN\_2020\_S-G2 |  | 186 | -0.43 | -2.28 | 0.000 | 0.000 | 0.004 | 864 | tags=46%, list=24%, signal=57% |
| 52 | SUVA\_G1S\_PMID\_31327527 |  | 20 | -0.65 | -2.26 | 0.000 | 0.000 | 0.006 | 1086 | tags=90%, list=30%, signal=128% |
| 53 | FELDMAN\_2020\_G1 |  | 85 | -0.46 | -2.25 | 0.000 | 0.000 | 0.007 | 846 | tags=49%, list=23%, signal=63% |
| 54 | RICHARDS\_NOWAKOWSKI\_SCIENCE\_2017\_EN\_PFC3\_UPREG |  | 116 | -0.45 | -2.25 | 0.000 | 0.000 | 0.007 | 905 | tags=47%, list=25%, signal=60% |
| 55 | RICHARDS\_NOWAKOWSKI\_SCIENCE\_2017\_IPC\_DIV2\_UPREG |  | 422 | -0.40 | -2.24 | 0.000 | 0.000 | 0.007 | 1305 | tags=58%, list=36%, signal=80% |
| 56 | RICHARDS\_NOWAKOWSKI\_SCIENCE\_2017\_MGE\_RG1\_UPREG |  | 161 | -0.42 | -2.24 | 0.000 | 0.000 | 0.007 | 666 | tags=37%, list=18%, signal=43% |
| 57 | RICHARDS\_NOWAKOWSKI\_SCIENCE\_2017\_IPC\_NEN1\_UPREG\_1 |  | 70 | -0.48 | -2.23 | 0.000 | 0.000 | 0.007 | 592 | tags=40%, list=16%, signal=47% |
| 58 | RICHARDS\_NOWAKOWSKI\_SCIENCE\_2017\_EN\_PFC1\_UPREG |  | 349 | -0.40 | -2.22 | 0.000 | 0.000 | 0.007 | 1080 | tags=49%, list=30%, signal=63% |
| 59 | RICHARDS\_NOWAKOWSKI\_SCIENCE\_2017\_NEN\_EARLY2\_UPREG |  | 204 | -0.41 | -2.21 | 0.000 | 0.000 | 0.007 | 1095 | tags=54%, list=30%, signal=73% |
| 60 | RICHARDS\_NOWAKOWSKI\_SCIENCE\_2017\_MGE\_RG2\_UPREG |  | 240 | -0.40 | -2.21 | 0.000 | 0.000 | 0.007 | 806 | tags=42%, list=22%, signal=50% |
| 61 | SEURAT\_CELLCYCLE |  | 62 | -0.49 | -2.20 | 0.000 | 0.000 | 0.007 | 1280 | tags=74%, list=35%, signal=113% |
| 62 | RICHARDS\_NOWAKOWSKI\_SCIENCE\_2017\_IN\_STR\_UPREG |  | 70 | -0.47 | -2.20 | 0.000 | 0.000 | 0.008 | 737 | tags=46%, list=20%, signal=56% |
| 63 | NB\_KI\_CLUSTER2 |  | 131 | -0.43 | -2.20 | 0.000 | 0.000 | 0.008 | 1012 | tags=56%, list=28%, signal=75% |
| 64 | RICHARDS\_ZHONG\_NATURE\_2018\_NPCS\_UPREG |  | 81 | -0.46 | -2.18 | 0.000 | 0.000 | 0.012 | 832 | tags=53%, list=23%, signal=67% |
| 65 | RICHARDS\_NOWAKOWSKI\_SCIENCE\_2017\_IN\_CTX\_MGE2\_UPREG |  | 107 | -0.43 | -2.17 | 0.000 | 0.000 | 0.016 | 1161 | tags=60%, list=32%, signal=85% |
| 66 | RICHARDS\_NOWAKOWSKI\_SCIENCE\_2017\_IPC\_DIV1\_UPREG |  | 317 | -0.39 | -2.15 | 0.000 | 0.000 | 0.020 | 852 | tags=41%, list=23%, signal=49% |
| 67 | RICHARDS\_NOWAKOWSKI\_SCIENCE\_2017\_NIN4\_UPREG |  | 65 | -0.46 | -2.14 | 0.000 | 0.000 | 0.021 | 574 | tags=35%, list=16%, signal=41% |
| 68 | FELDMAN\_2020\_NEURAL G0 |  | 243 | -0.39 | -2.14 | 0.000 | 0.000 | 0.021 | 634 | tags=34%, list=17%, signal=39% |
| 69 | RICHARDS\_NOWAKOWSKI\_SCIENCE\_2017\_IPC\_NEN3\_UPREG |  | 127 | -0.42 | -2.13 | 0.000 | 0.000 | 0.021 | 602 | tags=35%, list=17%, signal=40% |
| 70 | ALMSTEDT\_VGR\_DIFF |  | 363 | -0.37 | -2.11 | 0.000 | 0.000 | 0.035 | 818 | tags=39%, list=22%, signal=45% |
| 71 | RICHARDS\_NOWAKOWSKI\_SCIENCE\_2017\_IN\_CTX\_CGE1\_UPREG |  | 130 | -0.41 | -2.10 | 0.000 | 0.001 | 0.037 | 1003 | tags=49%, list=28%, signal=66% |
| 72 | WENG\_STEMNESS\_PMID\_30982771 |  | 16 | -0.63 | -2.07 | 0.000 | 0.001 | 0.053 | 346 | tags=50%, list=10%, signal=55% |
| 73 | GANDAL\_ALCOHOLADDICTION\_DN\_PMID29439242 |  | 30 | -0.55 | -2.07 | 0.000 | 0.001 | 0.055 | 747 | tags=47%, list=21%, signal=58% |
| 74 | ALMSTEDT\_RISK |  | 20 | -0.58 | -2.04 | 0.001 | 0.001 | 0.067 | 510 | tags=45%, list=14%, signal=52% |
| 75 | IVY\_IT |  | 31 | -0.52 | -2.02 | 0.001 | 0.001 | 0.079 | 880 | tags=55%, list=24%, signal=72% |
| 76 | DOROSZKO\_U3180MG\_CLUSTER\_3 |  | 237 | -0.37 | -2.02 | 0.000 | 0.001 | 0.079 | 1232 | tags=57%, list=34%, signal=80% |
| 77 | KRONA\_ROSEN\_WHITE\_MATTER\_INVASION |  | 130 | -0.39 | -2.00 | 0.000 | 0.001 | 0.092 | 1008 | tags=48%, list=28%, signal=65% |
| 78 | RICHARDS\_NOWAKOWSKI\_SCIENCE\_2017\_EN\_PFC2\_UPREG |  | 351 | -0.35 | -1.98 | 0.000 | 0.001 | 0.112 | 1120 | tags=47%, list=31%, signal=61% |
| 79 | DOROSZKO\_U3054MG\_CLUSTER\_4 |  | 274 | -0.36 | -1.98 | 0.000 | 0.001 | 0.113 | 1310 | tags=57%, list=36%, signal=83% |
| 80 | DOROSZKO\_U3013MG\_CLUSTER\_2 |  | 112 | -0.39 | -1.96 | 0.000 | 0.002 | 0.142 | 1229 | tags=61%, list=34%, signal=89% |
| 81 | FELDMAN\_2020\_G2-M |  | 143 | -0.37 | -1.94 | 0.000 | 0.002 | 0.171 | 618 | tags=30%, list=17%, signal=35% |
| 82 | DOROSZKO\_U3054MG\_CLUSTER\_8 |  | 240 | -0.35 | -1.92 | 0.000 | 0.003 | 0.221 | 1579 | tags=69%, list=43%, signal=113% |
| 83 | RICHARDS\_NOWAKOWSKI\_SCIENCE\_2017\_EN\_V1\_1\_UPREG |  | 348 | -0.34 | -1.90 | 0.000 | 0.003 | 0.255 | 1061 | tags=43%, list=29%, signal=55% |
| 84 | RICHARDS\_VERHAAK\_CANCERCELL\_2010\_CLASSICAL |  | 52 | -0.44 | -1.90 | 0.004 | 0.003 | 0.263 | 856 | tags=50%, list=24%, signal=64% |
| 85 | PARADA\_T2\_UP\_26461091 |  | 46 | -0.45 | -1.90 | 0.004 | 0.004 | 0.269 | 415 | tags=28%, list=11%, signal=31% |
| 86 | DALMO\_BARCODENUMBER\_UNPUB |  | 99 | -0.39 | -1.89 | 0.000 | 0.004 | 0.272 | 852 | tags=44%, list=23%, signal=56% |
| 87 | KLING\_GBM\_PHENO\_PMID\_27667176 |  | 83 | -0.39 | -1.87 | 0.002 | 0.004 | 0.325 | 613 | tags=33%, list=17%, signal=38% |
| 88 | DOROSZKO\_U3013MG\_CLUSTER\_3 |  | 54 | -0.43 | -1.85 | 0.005 | 0.005 | 0.370 | 821 | tags=43%, list=23%, signal=54% |
| 89 | DOROSZKO\_U3054MG\_CLUSTER\_10 |  | 104 | -0.37 | -1.82 | 0.001 | 0.006 | 0.458 | 356 | tags=21%, list=10%, signal=23% |
| 90 | RICHARDS\_NOWAKOWSKI\_SCIENCE\_2017\_MGE\_IPC1\_UPREG |  | 255 | -0.33 | -1.82 | 0.000 | 0.006 | 0.469 | 798 | tags=36%, list=22%, signal=43% |
| 91 | NB\_KI\_CLUSTER8 |  | 116 | -0.36 | -1.81 | 0.001 | 0.007 | 0.475 | 1579 | tags=75%, list=43%, signal=128% |
| 92 | RICHARDS\_NOWAKOWSKI\_SCIENCE\_2017\_NEN\_LATE\_UPREG |  | 144 | -0.35 | -1.80 | 0.000 | 0.007 | 0.508 | 606 | tags=28%, list=17%, signal=33% |
| 93 | RICHARDS\_CAHOY\_JNEUROSCI\_2014\_MOG\_POS |  | 259 | -0.32 | -1.78 | 0.000 | 0.008 | 0.575 | 1036 | tags=41%, list=28%, signal=53% |
| 94 | RICHARDS\_CAHOY\_JNEUROSCI\_2010\_ASTRO\_YOUNG |  | 365 | -0.31 | -1.78 | 0.000 | 0.009 | 0.592 | 875 | tags=37%, list=24%, signal=43% |
| 95 | RICHARDS\_NOWAKOWSKI\_SCIENCE\_2017\_NEN\_EARLY1\_UPREG |  | 261 | -0.32 | -1.75 | 0.001 | 0.011 | 0.678 | 1161 | tags=47%, list=32%, signal=64% |
| 96 | RICHARDS\_NOWAKOWSKI\_SCIENCE\_2017\_TRG\_UPREG |  | 490 | -0.30 | -1.72 | 0.000 | 0.014 | 0.775 | 585 | tags=24%, list=16%, signal=25% |
| 97 | DOROSZKO\_U3054MG\_CLUSTER\_5 |  | 55 | -0.39 | -1.71 | 0.014 | 0.015 | 0.792 | 519 | tags=29%, list=14%, signal=33% |
| 98 | RICHARDS\_NOWAKOWSKI\_SCIENCE\_2017\_EN\_V1\_2\_UPREG |  | 310 | -0.31 | -1.71 | 0.000 | 0.015 | 0.795 | 1073 | tags=42%, list=29%, signal=54% |
| 99 | RICHARDS\_NOWAKOWSKI\_SCIENCE\_2017\_NIN2\_UPREG |  | 214 | -0.31 | -1.68 | 0.001 | 0.018 | 0.855 | 754 | tags=31%, list=21%, signal=37% |
| 100 | ALMSTEDT\_ALK |  | 37 | -0.41 | -1.68 | 0.025 | 0.018 | 0.864 | 821 | tags=46%, list=23%, signal=59% |
| 101 | IVY\_LE |  | 26 | -0.44 | -1.67 | 0.026 | 0.018 | 0.870 | 880 | tags=50%, list=24%, signal=65% |
| 102 | GANDAL\_BIPOLAR\_DN\_PMID29439242 |  | 15 | -0.52 | -1.67 | 0.028 | 0.019 | 0.881 | 421 | tags=53%, list=12%, signal=60% |
| 103 | RICHARDS\_CAHOY\_JNEUROSCI\_2009\_NEURON |  | 354 | -0.29 | -1.66 | 0.000 | 0.019 | 0.893 | 1138 | tags=45%, list=31%, signal=59% |
| 104 | PARADA\_T1\_UP\_26461091 |  | 43 | -0.39 | -1.66 | 0.031 | 0.020 | 0.909 | 394 | tags=23%, list=11%, signal=26% |
| 105 | GANDAL\_SCHIZOPHRENIA\_UP\_PMID29439242 |  | 44 | -0.39 | -1.64 | 0.030 | 0.022 | 0.930 | 360 | tags=27%, list=10%, signal=30% |
| 106 | DOROSZKO\_U3054MG\_CLUSTER\_7 |  | 16 | -0.50 | -1.64 | 0.029 | 0.022 | 0.932 | 357 | tags=38%, list=10%, signal=41% |
| 107 | GAROFANO\_IAVARONE\_MTC\_SUBTYPE |  | 76 | -0.35 | -1.63 | 0.020 | 0.023 | 0.936 | 960 | tags=42%, list=26%, signal=56% |
| 108 | RICHARDS\_NOWAKOWSKI\_SCIENCE\_2017\_EN\_V1\_3\_UPREG |  | 318 | -0.29 | -1.62 | 0.000 | 0.026 | 0.952 | 1033 | tags=39%, list=28%, signal=49% |
| 109 | OLSEN\_MESENCHYMAL |  | 47 | -0.37 | -1.58 | 0.043 | 0.033 | 0.979 | 355 | tags=26%, list=10%, signal=28% |
| 110 | LARSSON\_STATE\_5 |  | 46 | -0.36 | -1.57 | 0.048 | 0.036 | 0.987 | 1180 | tags=52%, list=32%, signal=76% |
| 111 | RICHARDS\_CAHOY\_JNEUROSCI\_2011\_ASTRO\_MATURE |  | 210 | -0.29 | -1.57 | 0.005 | 0.036 | 0.987 | 1110 | tags=41%, list=31%, signal=56% |
| 112 | TF\_CENSUS\_TAIPALE |  | 358 | -0.28 | -1.56 | 0.000 | 0.036 | 0.987 | 741 | tags=27%, list=20%, signal=31% |
| 113 | RICHARDS\_CAHOY\_JNEUROSCI\_2008\_OLIGODENDROCYTE |  | 475 | -0.27 | -1.55 | 0.000 | 0.038 | 0.989 | 559 | tags=22%, list=15%, signal=23% |
| 114 | DOROSZKO\_U3054MG\_CLUSTER\_2 |  | 192 | -0.29 | -1.54 | 0.008 | 0.040 | 0.995 | 715 | tags=29%, list=20%, signal=34% |
| 115 | RICHARDS\_NOWAKOWSKI\_SCIENCE\_2017\_RG\_EARLY\_UPREG |  | 228 | -0.28 | -1.53 | 0.010 | 0.043 | 0.996 | 594 | tags=26%, list=16%, signal=29% |
| 116 | NB\_KI\_CLUSTER17 |  | 87 | -0.32 | -1.53 | 0.038 | 0.044 | 0.996 | 815 | tags=37%, list=22%, signal=46% |
| 117 | RICHARDS\_NOWAKOWSKI\_SCIENCE\_2017\_MURAL\_UPREG |  | 300 | -0.27 | -1.48 | 0.006 | 0.058 | 1.000 | 631 | tags=26%, list=17%, signal=29% |
| 118 | DOROSZKO\_U3013MG\_CLUSTER\_1 |  | 24 | -0.41 | -1.48 | 0.088 | 0.059 | 1.000 | 805 | tags=46%, list=22%, signal=58% |
| 119 | NB\_KI\_CLUSTER23 |  | 116 | -0.29 | -1.48 | 0.029 | 0.060 | 1.000 | 651 | tags=28%, list=18%, signal=34% |
| 120 | VANGRONINGEN\_MES |  | 217 | -0.27 | -1.47 | 0.013 | 0.062 | 1.000 | 815 | tags=31%, list=22%, signal=37% |
| 121 | NB\_KI\_CLUSTER18 |  | 52 | -0.33 | -1.45 | 0.072 | 0.071 | 1.000 | 336 | tags=23%, list=9%, signal=25% |
| 122 | RICHARDS\_ZHONG\_NATURE\_2018\_MICROGLIA\_UPREG |  | 119 | -0.28 | -1.44 | 0.045 | 0.071 | 1.000 | 624 | tags=29%, list=17%, signal=33% |
| 123 | ALMSTEDT\_11Q\_MSIGDB |  | 132 | -0.27 | -1.37 | 0.079 | 0.109 | 1.000 | 654 | tags=25%, list=18%, signal=29% |
| 124 | PARADA\_T1\_DN\_26461091 |  | 21 | -0.38 | -1.34 | 0.157 | 0.126 | 1.000 | 1138 | tags=57%, list=31%, signal=83% |
| 125 | NB\_KI\_CLUSTER6 |  | 144 | -0.25 | -1.33 | 0.110 | 0.135 | 1.000 | 1680 | tags=67%, list=46%, signal=119% |
| 126 | DOROSZKO\_U3054MG\_CLUSTER\_3 |  | 98 | -0.27 | -1.31 | 0.127 | 0.144 | 1.000 | 749 | tags=31%, list=21%, signal=38% |
| 127 | GANDAL\_IBD\_UP\_PMID29439242 |  | 45 | -0.31 | -1.31 | 0.146 | 0.145 | 1.000 | 355 | tags=20%, list=10%, signal=22% |
| 128 | DOROSZKO\_U3054MG\_CLUSTER\_6 |  | 46 | -0.30 | -1.29 | 0.189 | 0.164 | 1.000 | 1327 | tags=59%, list=36%, signal=91% |
| 129 | GANDAL\_BIPOLAR\_UP\_PMID29439242 |  | 42 | -0.30 | -1.26 | 0.219 | 0.189 | 1.000 | 560 | tags=29%, list=15%, signal=33% |
| 130 | WANG\_CLASSICAL\_PMID\_28697342 |  | 16 | -0.38 | -1.24 | 0.222 | 0.200 | 1.000 | 1558 | tags=75%, list=43%, signal=131% |
| 131 | RICHARDS\_CAHOY\_JNEUROSCI\_2013\_OL\_MYEL |  | 427 | -0.22 | -1.23 | 0.099 | 0.212 | 1.000 | 1171 | tags=38%, list=32%, signal=49% |
| 132 | NB\_KI\_CLUSTER14 |  | 100 | -0.24 | -1.22 | 0.203 | 0.225 | 1.000 | 1071 | tags=39%, list=29%, signal=54% |
| 133 | GANDAL\_AUTISM\_UP\_PMID29439242 |  | 43 | -0.28 | -1.16 | 0.283 | 0.293 | 1.000 | 247 | tags=14%, list=7%, signal=15% |
| 134 | GANDAL\_ALCOHOLADDICTION\_UP\_PMID29439242 |  | 59 | -0.25 | -1.15 | 0.293 | 0.295 | 1.000 | 678 | tags=25%, list=19%, signal=31% |
| 135 | NB\_KI\_CLUSTER16 |  | 57 | -0.26 | -1.14 | 0.314 | 0.304 | 1.000 | 156 | tags=12%, list=4%, signal=13% |
| 136 | RICHARDS\_NOWAKOWSKI\_SCIENCE\_2017\_U3\_UPREG |  | 279 | -0.20 | -1.12 | 0.290 | 0.334 | 1.000 | 869 | tags=28%, list=24%, signal=34% |
| 137 | IVY\_CTMVP |  | 53 | -0.25 | -1.11 | 0.350 | 0.340 | 1.000 | 508 | tags=23%, list=14%, signal=26% |
| 138 | GANDAL\_DEPRESSION\_DN\_PMID29439242 |  | 24 | -0.30 | -1.09 | 0.382 | 0.372 | 1.000 | 673 | tags=38%, list=18%, signal=46% |
| 139 | RICHARDS\_NOWAKOWSKI\_SCIENCE\_2017\_U1\_UPREG |  | 495 | -0.18 | -1.04 | 0.417 | 0.446 | 1.000 | 634 | tags=19%, list=17%, signal=20% |
| 140 | GANDAL\_IBD\_DN\_PMID29439242 |  | 16 | -0.30 | -1.00 | 0.456 | 0.489 | 1.000 | 792 | tags=38%, list=22%, signal=48% |
| 141 | RICHARDS\_NOWAKOWSKI\_SCIENCE\_2017\_CHOROID\_UPREG |  | 209 | -0.17 | -0.92 | 0.636 | 0.616 | 1.000 | 461 | tags=13%, list=13%, signal=14% |
| 142 | DOROSZKO\_U3180MG\_CLUSTER\_6 |  | 26 | -0.24 | -0.87 | 0.613 | 0.679 | 1.000 | 294 | tags=15%, list=8%, signal=17% |
| 143 | NB\_KI\_CLUSTER7 |  | 60 | -0.12 | -0.53 | 0.965 | 0.986 | 1.000 | 2681 | tags=88%, list=74%, signal=330% |
| 144 | NB\_KI\_CLUSTER24 |  | 41 | -0.12 | -0.50 | 0.969 | 0.988 | 1.000 | 1210 | tags=34%, list=33%, signal=51% |
| 145 | RICHARDS\_VERHAAK\_CANCERCELL\_2010\_NEURAL |  | 22 | -0.14 | -0.49 | 0.952 | 0.983 | 1.000 | 1444 | tags=50%, list=40%, signal=82% |
Table: Gene sets enriched in phenotype **na **[plain text format]****
